# The impact of challenge-hindrance research stress on burnout among healthcare workers: the moderating role of perceived organizational support

**DOI:** 10.3389/fpubh.2025.1618077

**Published:** 2025-09-08

**Authors:** Xianglan Chen, Chengping Jian, Xiaohong Shen, Ruixue Liu, Yuan Pu, Shuhua Wang

**Affiliations:** ^1^Department of Scientific Research Management, The Sixth Affiliated Hospital of Kunming Medical University, Yuxi, Yunnan, China; ^2^Department of Ophthalmology, Mianyang Central Hospital, Mianyang, Sichuan, China

**Keywords:** challenge research stress, hindrance research stress, burnout, perceived organizational support, moderating role

## Abstract

**Objective:**

This study explores the impact of challenge-hindrance research stress on burnout among healthcare workers and examines the moderating role of perceived organizational support (POS). The findings aim to provide suggestions for alleviating burnout in healthcare workers.

**Methods:**

Data were collected using the Demographic Questionnaire, Burnout Scale, Research Stress Scale, and Perceived Organizational Support Scale. Relationships and moderation effects were analyzed via SPSS and PROCESS Macro.

**Results:**

Both challenge research stress (*r* = 0.156, *p* < 0.05) and hindrance research stress (*r* = 0.403, *p* < 0.01) were significantly positively correlated with burnout. Linear regression revealed that POS significantly negatively moderated the relationship between hindrance research stress and burnout (*β* = −0.137, *p* < 0.05). PROCESS analysis indicated that hindrance research stress was significantly associated with low POS (*β* = 0.460, *p* < 0.001), but not significant at high POS (*β* = 0.159, *p* > 0.05). No significant moderating role of POS was found between challenge research stress and burnout.

**Conclusion:**

Medical institutions should focus on reducing hindrance research stress while implementing organizational support interventions, including optimized resource distribution and procedural streamlining to mitigate burnout. Regarding challenge research stress, strategies should emphasize the enhancement of individual self-management capabilities.

## 1 Introduction

Burnout refers to a series of negative emotional responses in individuals due to prolonged work stress, including emotional exhaustion, negative attitudes toward work, and a reduced sense of personal achievement (1). Burnout, a response to high work stress, manifests through three dimensions: emotional exhaustion, depersonalization, and reduced personal accomplishment. Burnout is characterized by emotional exhaustion and a lack of emotional resources; negative and detached responses toward others, leading to a loss of idealism; and a decline in work ability and performance (2).

In the United States, more than half of healthcare workers experience burnout during their careers (3). A systematic review in China reported burnout rates among healthcare workers ranging from 66.5 to 87.8% (4). Some studies show that the burnout rate among grassroots healthcare workers exceeds 90% in China. Additionally, research indicates that public health emergencies exacerbate burnout among healthcare workers (5, 6). In January 2022, the World Health Organization (WHO) included “burnout” as a formal disease for the first time in the 11th edition of the International Classification of Diseases.

Healthcare workers face a high risk of burnout due to excessive work stress, occupational hazards, and strained doctor-patient relationships (7). Healthcare workers have become a high-risk group for burnout, which has become a widespread global public health issue. Furthermore, the high incidence of burnout not only affects the physical and mental health of healthcare workers (8), but also has adverse effects on medical quality, work efficiency, patient experience, and healthcare costs (9, 10). Currently, there is an urgent need to implement effective interventions to alleviate burnout among healthcare workers.

Researchers worldwide have long studied healthcare workers’ mental health, focusing on burnout correlations with specific issues. For example, studies have examined the imbalance between effort and reward (11), job satisfaction (12), depression (13), occupational stress (14), sleep quality (15), self-rated health status (16). Overall, the factors influencing burnout among healthcare workers can be categorized into four major aspects: individual factors, work factors, social factors, and organizational factors (17).

Organizational support, as an important resource, helps healthcare workers reduce work stress and alleviate burnout (18). Moreover, it can only form POS when it is recognized by the individual. POS refers to the employee’s overall perception of how the organization values their contributions and cares about their wellbeing (19). POS can effectively promote positive individual outcomes, such as physical and mental health, work engagement, and job satisfaction (20).

It is worth noting that large hospitals typically bear heavy clinical teaching and research responsibilities. Healthcare workers face immense work and research pressures, with work stress exacerbating burnout among them (21). Challenge research stress refers to demands that, though demanding, are perceived as opportunities for skill development and achievement. In contrast, hindrance research stress involves tasks or environments that hinder goal achievement, reduce enthusiasm, and cause frustration. Cavanaugh’s two-dimensional model of stress theory suggests that challenge stress can enhance an individual’s work motivation, whereas hindrance stress decreases it.

In practice, the heavy research tasks and strict evaluation systems in large hospitals contribute to significant research pressure on healthcare workers, which may exacerbate burnout. There have been interventions at the organizational level (22), such as work control (23), strengthening social support (24), focusing on psychological capital (25), establishing a humanitarian care mechanism (26), and improving the work environment (27), to alleviate burnout among healthcare workers.

However, few studies have examined how challenge and hindrance research stress differentially impact burnout, or how POS moderates these relationships. Moreover, the underlying mechanisms need further exploration. Although existing studies have revealed the significant intervention or moderating role of POS (28–30), its moderating role in the relationship between research pressure and burnout among healthcare workers has not been fully validated.

This study investigates the effects of challenge and hindrance research stress on burnout among healthcare workers. Additionally, it examines how POS moderates these relationships. The goal is to provide recommendations for hospital and healthcare management on research management and burnout intervention, improving the mental health of healthcare workers and alleviating burnout.

## 2 Materials and methods

### 2.1 Sample source

From November to December 2024, a convenience sampling method was used to recruit 232 healthcare workers from the Sixth Affiliated Hospital of Kunming Medical University. Data were collected through electronic questionnaires. A total of 232 questionnaires were returned. After excluding 22 invalid responses, 210 valid questionnaires remained, yielding an effective response rate of 91%.

Inclusion criteria were as follows: (1) being on duty during the survey period; (2) engagement in clinical, medical technology, nursing, or administrative work; (3) provision of informed consent and voluntary participation. Exclusion criteria included: (1) completion time > 20 min or <2 min; (2) healthcare workers on sick leave or maternity leave during the study period; (3) regular completion of questionnaires.

The cover page of the questionnaire detailed the purpose, procedures, and confidentiality measures of the study. Participants provided informed consent, and all data were anonymized for privacy protection. This study was approved by the Medical Ethics Committee of the Sixth Affiliated Hospital of Kunming Medical University (approval number: 2024kmykdx6f075).

To enhance data reliability and representativeness, the research team carefully managed the questionnaire distribution and collection process, ensuring participants represented diverse departments and job levels. Flexible timing was provided to accommodate healthcare workers’ busy schedules. Data monitoring was used to exclude invalid questionnaires and ensure data quality.

### 2.2 Measurement tools

#### 2.2.1 Demographic questionnaire

The General Information Survey was designed by the researchers. It collected personal information, including gender (male, female), age (20–29 years, 30–39 years, 40–49 years, 50–59 years), education level (associate’s degree, bachelor’s degree, master’s degree, doctorate or above), and health status (healthy, sub-healthy, unhealthy). Professional information was also collected, including job position (clinical, nursing, medical technology, administrative management) and average daily working hours (5–8 h, 8–10 h, 10–12 h, more than 12 h).

#### 2.2.2 Burnout scale

The Chinese version of the Burnout Scale was adapted from the Maslach Burnout Inventory–General Survey (MBI–GS) to measure burnout. The scale includes three dimensions: emotional exhaustion (5 items), cynicism (4 items), and reduced personal accomplishment (6 items). A 7-point Likert scale was used, ranging from 0 (“never”) to 6 (“very frequently”). Emotional exhaustion and cynicism are scored positively, while reduced personal accomplishment is scored negatively. The three dimensions had Cronbach’s *α* coefficients of 0.88, 0.83, and 0.82.

#### 2.2.3 Research stress scale

The Chinese version of the Research Stress Scale includes two dimensions: challenge research stress (6 items) and hindrance research stress (8 items). Challenge research stress refers to stress that stimulates an individual’s initiative. In contrast, hindrance research stress refers to stress that negatively affects performance and causes frustration. A 5-point Likert scale was used, ranging from 1 (“strongly disagree”) to 5 (“strongly agree”). Higher scores indicate greater research stress. The Cronbach’s *α* coefficients were 0.88 and 0.84 for each dimension.

#### 2.2.4 Perceived organizational support scale

The Chinese version of the POS Scale includes two dimensions: emotional support (8 items) and instrumental support (6 items). Emotional support refers to the emotional encouragement and support provided by the organization. Instrumental support refers to the resources provided by the organization to help employees accomplish their tasks. A 5-point Likert scale was used, ranging from 1 (“strongly disagree”) to 5 (“strongly agree”). Higher scores indicate stronger perceived POS. A total Cronbach’s *α* coefficient of 0.92 was obtained.

### 2.3 Research framework and data analysis

This study was based on the Job Demands-Resources (JD-R) model as its theoretical foundation. This model can well explain the impact of job demands and resources on employees’ physical and mental health. In this study, research stress is regarded as a part of job demands, perceived organizational support as a part of job resources, and burnout as the result of the interaction between job demands and resources.

The data were analyzed using IBM SPSS Statistics (Version 27.0). Harman’s single-factor test was employed to check for common method bias (31). Exploratory factor analysis was conducted on all scale items to analyze the number of factors with eigenvalues greater than 1 and the percentage of variance explained by these factors.

Burnout severity was categorized based on total scores, with higher scores indicating more severe burnout. The detection rate of burnout and the scores of each variable were described. Continuous data with normal distribution were presented as mean ± standard deviation (M ± SD), while categorical data were described using percentages (%). Pearson correlation analysis was used to examine the linear relationship between challenge research stress, hindrance research stress, POS, and burnout.

To test the moderating role of POS (28, 32), hierarchical regression analysis was conducted. For the analysis, burnout was set as the dependent variable, with challenge research stress and hindrance research stress as independent variables, and POS as the moderator variable. The control variables include age, gender, education level, job position, average daily working hours, and self-rated health status. All continuous variables were standardized before performing the regression analysis.

PROCESS Macro (Model 1, Hayes) was used to test the significance of the moderated model. This tool facilitated the specific analysis and graphical representation of the moderating role of POS. Simple slope analysis was conducted to probe the relationship at high (+1 SD) and low (−1 SD) levels of POS. All statistical tests were two-sided, with a significance level set at *p* < 0.05.

**Figure 1:**
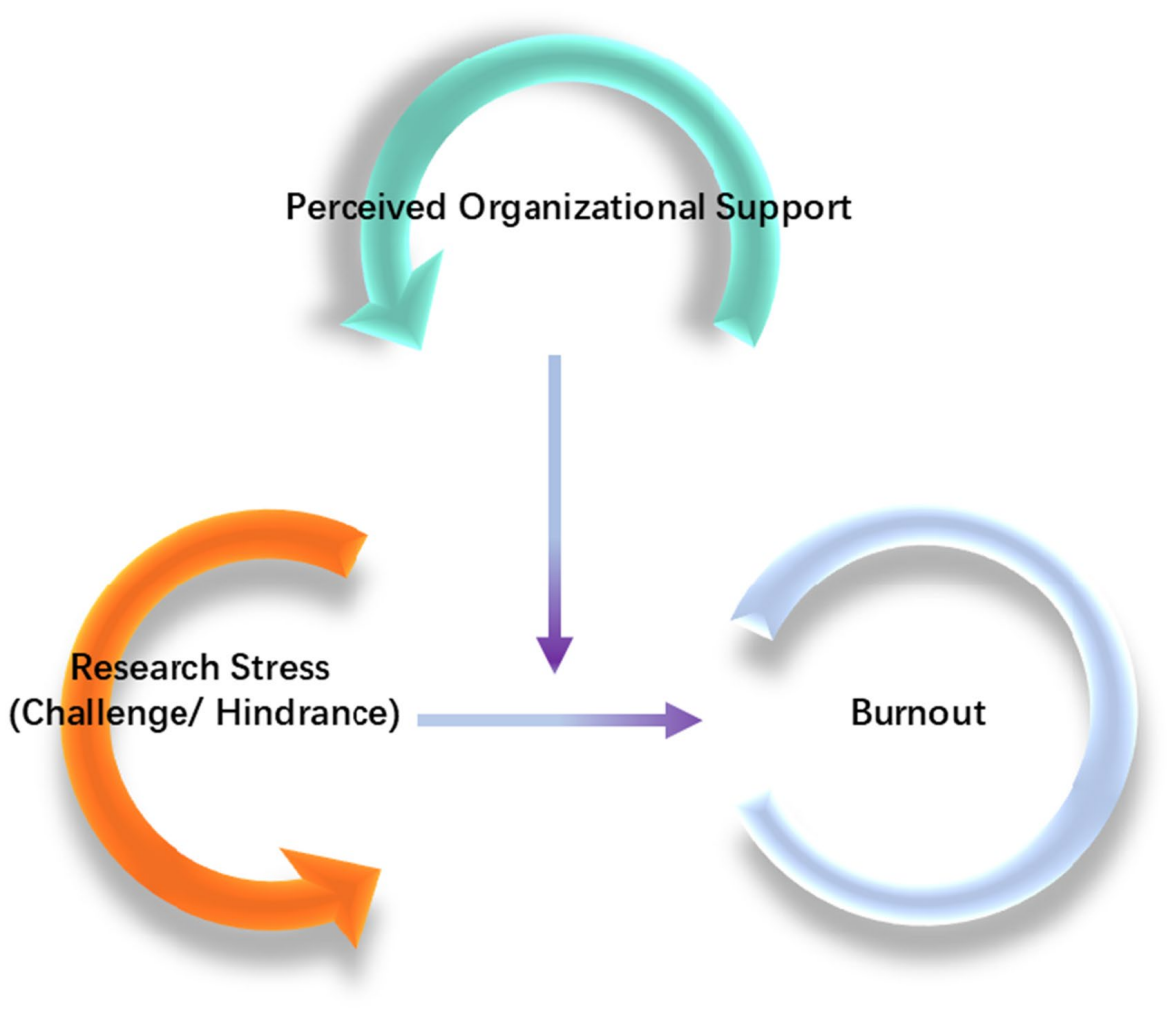
Theoretical framework.

It is hypothesized that both challenge research stress and hindrance research stress affect burnout. Additionally, POS is hypothesized to moderate the relationship between research stress and burnout, potentially influencing burnout either positively or negatively. The theoretical framework is presented in Figure 1.

**Figure 2:**
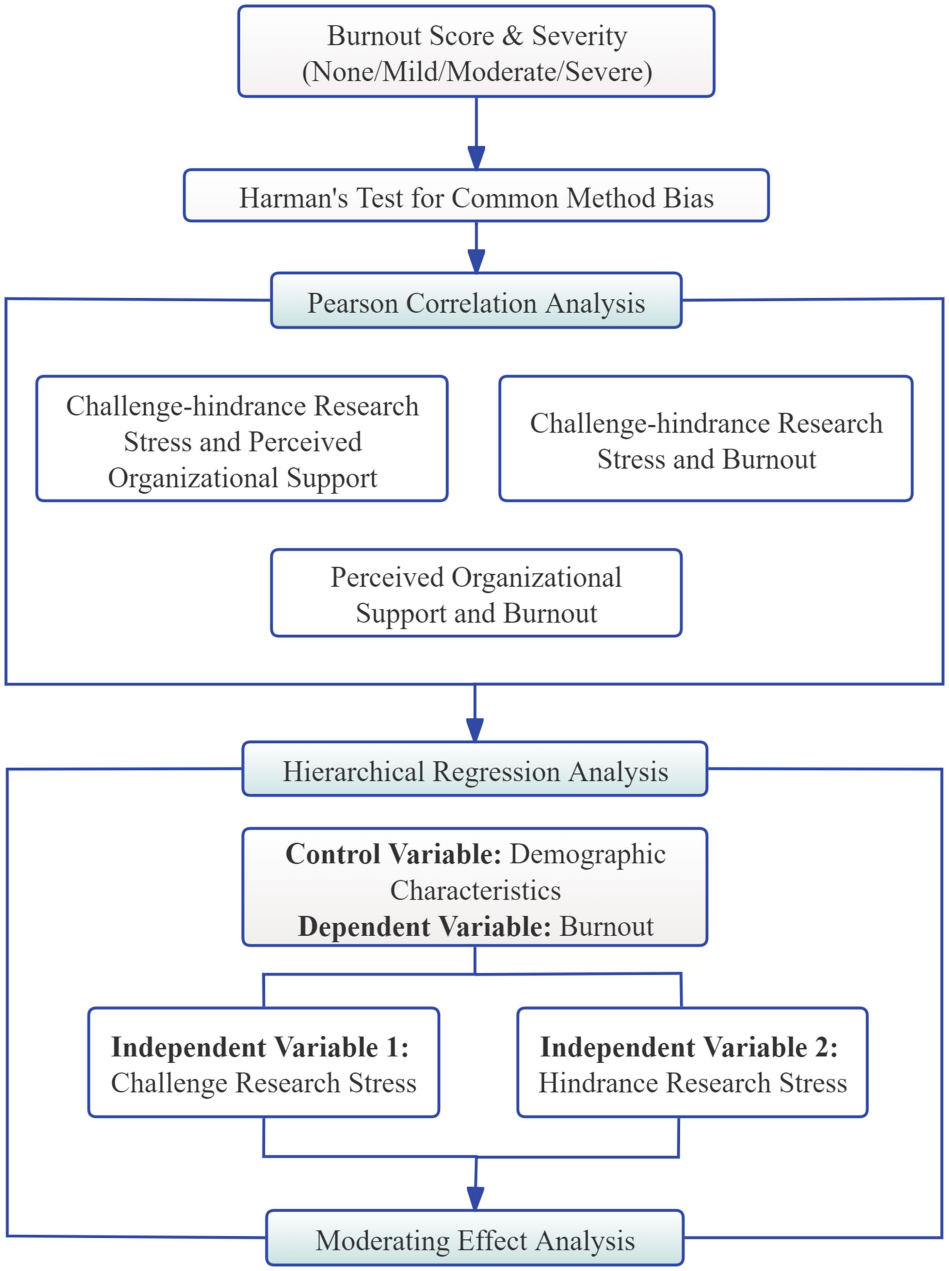
Technical flowchart.

The technical flowchart of the statistical methodology is illustrated in Figure 2. The statistical analysis was carried out in three steps. First, Pearson correlation analysis was used to explore the relationships among research stress, burnout, and POS. Second, hierarchical linear regression was conducted with burnout as the dependent variable, two types of research stress as independent variables, and demographic and work-related factors as controls. Finally, the moderation effect of POS was tested via the PROCESS macro (Model 1).

## 3 Results

### 3.1 Common method bias test

To ensure the reliability of the data, Harman’s single-factor test was first used to assess common method bias. This test performs exploratory factor analysis on all the survey items to examine whether a single factor explains most of the variance. If the first factor explains more than 40% of the variance, it indicates the presence of common method bias, which could affect the validity of the research results. In this study, the first factor explained 32.17% of total variance, below the 40% threshold, indicating no critical common method bias.

### 3.2 Demographic characteristics

Among the 210 participants, 58 were male (27.6%) and 152 were female (72.4%). The detailed information was shown in Table 1.

**Table 1:** Demographic characteristics of participants (*N* = 210).

Item	Count	Percentage
Gender		
Male	58	27.6%
Female	152	72.4%
Age		
20–29 years	44	21%
30–39 years	94	44.8%
40–49 years	53	25.2%
50–59 years	19	9%
Education level		
Associate’s degree	5	2.4%
Bachelor’s degree	111	52.9%
Master’s degree	92	43.8%
Doctoral degree or higher	2	0.9%
Job position		
Clinical doctors	83	39.5%
Nursing staff	73	34.8%
Medical technicians	34	16.2%
Administrative staff	20	9.5%
Daily working hours		
5–8 h	26	12.4%
8–10 h	131	62.4%
10–12 h	38	18.1%
More than 12 h	15	7.1%
Self-rated health status		
Healthy	60	28.6%
Sub-healthy	140	66.7%
Unhealthy	10	4.7%

### 3.3 Burnout situation

The total score of the burnout scale is computed using the following formula: Emotional exhaustion score × 0.4 + Cynicism score × 0.3 + Reduced personal accomplishment score × 0.3. Burnout severity was categorized as follows: None (<1.5), Mild [1.5–3.0), Moderate [3.0–5.0), and Severe (≥5.0) (33).

**Figure 3:**
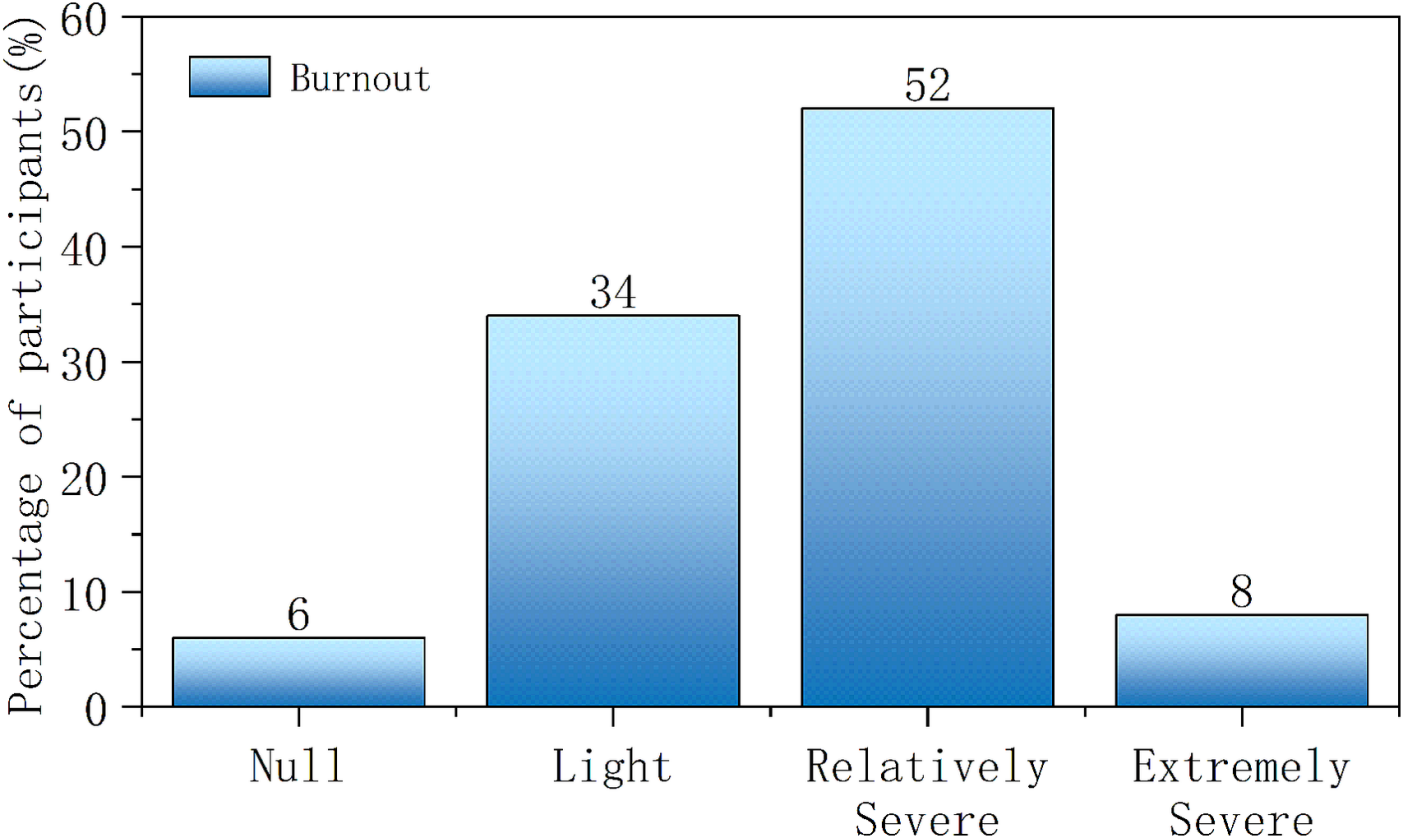
Burnout status.

The statistical results show that the burnout prevalence among medical staff was 94.29%. There are 12 individuals with no burnout (5.71%), 71 individuals with mild burnout (33.81%), 109 individuals with moderate burnout (51.91%), and 18 individuals with severe burnout (8.57%), as shown in Figure 3.

The total burnout score was 3.33 ± 1.17, with the emotional exhaustion dimension scoring 4.23 ± 1.70, cynicism dimension scoring 2.87 ± 1.56, and reduced personal accomplishment dimension scoring 2.59 ± 1.25, as shown in Table 2.

**Table 2:** Scores of each dimension of burnout.

Variable	Mean (x̄)	Standard Deviation (SD)
Emotional exhaustion	4.23	1.70
Cynicism	2.87	1.56
Reduced personal accomplishment	2.59	1.25
Total burnout score	3.33	1.17

### 3.4 Pearson correlation analysis of challenge and hindrance research stress, POS and burnout

The scores for challenge research stress, hindrance research stress, and POS were 3.28 ± 0.79, 3.26 ± 0.88, and 3.41 ± 0.65, while the total burnout score was 3.33 ± 1.17.

Pearson correlation analysis results revealed that challenge research stress was significantly positively correlated with the total burnout score (*r* = 0.156, *p* < 0.05), hindrance research stress was significantly positively correlated with the total burnout score (*r* = 0.403, *p* < 0.01), and POS was significantly negatively correlated with the total burnout score (*r* = −0.507, *p* < 0.01). Additionally, challenge research stress was significantly negatively correlated with POS (*r* = −0.229, *p* < 0.01), and hindrance research stress was significantly negatively correlated with POS (*r* = −0.394, *p* < 0.01). As shown in Table 3.

**Table 3:** Pearson correlation analysis results.

Variable	M ± SD	Challenge research stress	Hindrance research stress	POS	Total burnout score
Challenge research stress	3.28 ± 0.79	1			
Hindrance research stress	3.26 ± 0.88	0.421^**^	1		
POS	3.65 ± 0.76	−0.229^**^	−0.394^**^	1	
Total burnout score	3.33 ± 1.17	0.156^*^	0.403^**^	−0.507^**^	1

*p < 0.05, **p < 0.01.

In summary, both challenge and hindrance research stress were positively correlated with burnout and negatively correlated with POS. POS was negatively correlated with burnout.

### 3.5 Moderating role of POS between research stress and burnout

To address potential collinearity issues, all independent variables, the moderator variable, and other continuous variables were standardized using z-scores. Some demographic factors that may affect burnout in previous studies were selected as control variables, including age, gender, education level, job position, average daily working hours, and self-rated health status. Burnout was set as the dependent variable, while challenge research stress and hindrance research stress were independent variables, and POS was the moderating variable.

Hierarchical regression analysis was used to test the moderating role of POS between challenge-hindrance research stress and burnout. In the first step, only control variables were included. In the second step, the main effects of challenge research stress and POS were added. In the third step, the interaction effect of POS and challenge research stress was included.

The results are shown in M1, M2, and M3 in Table 4. The main effects of challenge research stress and POS were not significant (*p* > 0.05), and the interaction effect between challenge research stress and POS was also not significant (*p* > 0.05). This suggests that POS may not significantly moderate the relationship between challenge research stress and burnout.

**Table 4:** Hierarchical regression analysis results.

Variable	Burnout					
	M1	M2	M3	M4	M5	M6
Control variables						
Gender	−0.006	0.039	0.033	−0.006	0.026	0.008
Age	−0.222^**^	−0.195^**^	−0.184^**^	−0.222^***^	−0.178^**^	−0.181^**^
Education level	0.110	0.122*	0.120*	0.110	0.143^*^	0.134^*^
Job position	−0.010	−0.024	−0.023	−0.010	−0.014	−0.009
Daily working hours	0.162^*^	0.068	0.073	0.162^*^	0.065	0.058
Self-rated health status	0.195^**^	0.191^**^	0.202^***^	0.195^**^	0.168^**^	0.182^**^
Independent variables						
Challenge research stress		0.036	0.005			
Hindrance research stress					0.206^***^	0.199^**^
Moderating variable						
POS		−0.477^***^	−0.474^***^		−0.406^***^	−0.388^***^
Interaction terms						
Challenge research stress × POS			−0.110			
Hindrance research stress × POS						−0.137^*^
*R* ^2^	0.145	0.371	0.382	0.145	0.405	0.423
△*R*^2^	0.119	0.346	0.354	0.119	0.381	0.397
*F*	5.716^***^	14.827^***^	13.733^***^	5.716^***^	17.082^***^	16.264^***^

*p < 0.05, **p < 0.01, ***p < 0.001.

Using the same method, the moderating role of POS between hindrance research stress and burnout was tested, with results presented in M4, M5, and M6 in Table 4. The main effect of hindrance research stress was significant (*β* = 0.206, *p* < 0.001), the main effect of POS was significant (*β* = −0.406, *p* < 0.001), and the interaction effect between hindrance research stress and POS was significant (*β* = −0.137, *p* < 0.05). This indicates that POS significantly negatively moderates the relationship between hindrance research stress and burnout.

**Figure 4:**
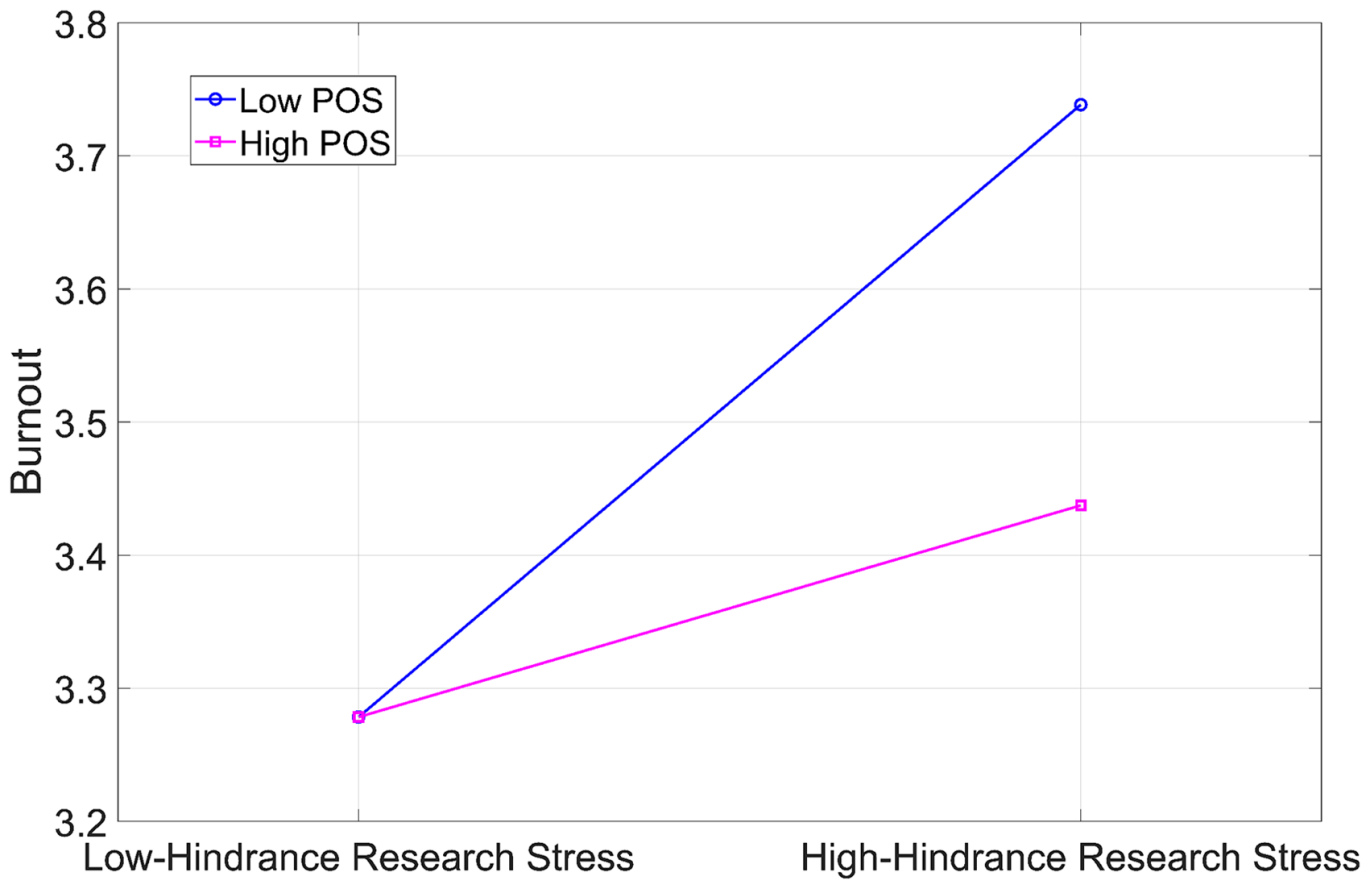
Moderating role of POS on hindrance research stress and burnout.

As shown in Figure 4 and Table 5, under low POS (−1 SD), hindrance research stress significantly predicted burnout (*β* = 0.460, *p* < 0.001), with a 95% confidence interval of [0.257, 0.664]. Under high POS (+1 SD), hindrance research stress did not significantly predict burnout (*β* = 0.159, *p* > 0.05), with a 95% confidence interval of [−0.054, 0.372]. Simple slope analysis confirmed that POS buffers the link between hindrance research stress and burnout: the relationship was significant at low levels of POS but not at high levels.

**Table 5:** Moderating role of POS on hindrance research stress and burnout.

Variable	Effect	se	*t*	*p*	LLCI	ULCI
Low POS	0.460	0.103	4.459	0.000	0.257	0.664
High POS	0.159	0.108	1.471	0.143	−0.054	0.372

LLCI, Lower Limit of Confidence Interval; ULCI, Upper Limit of Confidence Interval.

These findings suggest that as POS increases, the association between hindrance research stress and burnout is attenuated. This indicates that high POS may mitigate the impact of hindrance research stress on burnout.

## 4 Discussion

This study explores the impact of challenge and hindrance research stress on burnout among healthcare workers. Both types of stress are significantly positively correlated with burnout. While challenge research stress may stimulate work motivation and yield certain positive outcomes, prolonged exposure to high levels of such stress can still contribute to increased burnout. Conversely, hindrance research stress often characterized by insurmountable difficulties or obstacles, not only directly increases the work burden but also diminishes the psychological wellbeing of healthcare workers, potentially exacerbating burnout. These findings are consistent with Cavanaugh’s two-dimensional stress theory and other related research (34, 35), emphasizing the importance of differentiated management for various types of stress.

This study validated the significant moderating role of POS on the relationship between stress and burnout (36, 37). Specifically, when healthcare professionals perceive higher organizational support, the positive predictive effect of hindrance research stress on job burnout is weakened. In contrast, under conditions of low organizational support, the impact of hindrance research stress on job burnout is more significant. This suggests that organizational support, as an important resource, may effectively reduce the impact of hindrance research stress on burnout. The positive role of POS should be emphasized (38, 39), as it plays a significant role in managing both burnout and research stress.

However, no significant moderating effect of POS was found between challenge research stress and burnout. This may be because challenge stress has inherent positive valence, which reduces the need for external support. Moreover, individuals dealing with challenge demands tend to rely more on personal resources such as self-efficacy and intrinsic motivation (40). Additionally, the buffering effect of psychological capital can dilute POS’s influence (41). These findings emphasize the significance of self-management for challenge stress. It is crucial to maintain challenge stress at optimal levels to promote medical innovation while avoiding overload.

The research was based on a unidirectional framework in which research stress affects burnout. Attention should be paid to the possibility of potential reverse causal paths. Healthcare workers with higher levels of burnout may perceive research stress more acutely. When highly burned-out, their psychological and physiological resources are over-consumed, and their tolerance for stress decreases. As a result, they may find the tasks brought by challenge research stress more difficult and the obstacles from hindrance research stress harder to overcome. A study showed that burnout levels significantly predicted an increase in the perception of hindrance stress (42). Meanwhile, healthcare workers with high burnout may view routine job demands as more obstructive, indicating that the causal relationship may be bidirectional. Addressing burnout requires a multifaceted approach incorporating both individual coping strategies and organizational support systems (43, 44).

This study has significant practical implications. On one hand, healthcare institutions should strengthen the classification management of research stress among healthcare workers. This can be achieved by enhancing POS (45), reasonably allocating research tasks, and optimizing performance evaluation systems (46). On the other hand, healthcare institutions should focus on enhancing healthcare workers’ perception of organizational support. This can be achieved by reducing work pressure (47), strengthening personal and organizational support systems (48), and providing continuous professional development opportunities. These measures will help healthcare workers better cope with stress, improve their psychological wellbeing, and enhance their overall mental health satisfaction and levels (49, 50).

Although this study provides valuable insights, there are still some limitations. First, the study uses a cross-sectional survey design, and causal inferences need further validation. Second, the sample is drawn from a single hospital, and the sample size is limited, which may restrict the generalizability of the findings. Finally, no power analysis was conducted to determine the sample size before data collection. Low statistical power might have affected the detection of the significant predictive effect of challenge research stress on burnout despite verifying a correlation between these constructs.

Future research can focus on the following aspects: (1) Adopt a longitudinal research design for long-term follow-up of the subjects, aiming to verify the causal relationship between research stress and burnout. (2) Expand the sample scope by including samples from diverse geographical regions, various hospital types, and different organizational structures. This can enhance the sample’s diversity and representativeness. (3) Conduct a power analysis before commencing the research to calculate the minimum sample size required for sufficient statistical power. (4) Explore potential mediating or moderating variables, such as psychological capital, social support, and effort-reward imbalance, to gain a more comprehensive understanding of the underlying mechanism between research stress and burnout.

## 5 Conclusion

This study explored the impacts of challenge and hindrance research stress on the burnout of healthcare workers. The findings revealed that both types of stress were significantly and positively correlated with burnout. POS significantly moderated the relationship between hindrance research stress and burnout. Under low POS conditions, the effect of hindrance stress on burnout was more pronounced. High POS could mitigate the negative impact of hindrance stress on burnout.

Additionally, no significant moderating effect of POS was found on the relationship between challenge research stress and burnout, possibly due to the unique work patterns of medical researchers. When facing high-challenge demands such as research innovation, researchers rely more on personal resources and research capabilities.

The results indicate that hindrance research stress is a key factor leading to burnout among healthcare workers, while POS can alleviate its negative effects. These findings offer important practical implications for medical institutions. They should reduce burnout by enhancing POS and improving employees’ perception of support. Meanwhile, although challenge stress has certain positive incentives, improper management can still exacerbate burnout, suggesting the need to establish appropriate regulatory mechanisms and strengthen individual coping abilities.

Organizations should establish differentiated support systems for different types of stress. For hindrance stress, priority should be given to improving the procedural justice guarantee mechanism, such as optimizing the ethical review process and simplifying administrative procedures to reduce unnecessary bureaucratic burdens on researchers. For challenge stress, targeted resources like special research funds and professional training opportunities should be allocated to fully stimulate researchers’ innovation potential by enhancing their capabilities.

In conclusion, this study provides a theoretical basis for the research management practices of hospitals and health management departments, highlighting the importance of organizational support, research management optimization, and individual research stress regulation. Future research can explore multi-dimensional burnout intervention measures, including implementing classified management strategies for research stress, strengthening organizational support, providing psychological counseling services, and improving the performance appraisal system. These strategies are expected to enhance healthcare workers’ job satisfaction and mental health, ultimately alleviating the widespread burnout problem in the medical industry.

## Data Availability

The raw data supporting the conclusions of this article will be made available by the authors without undue reservation.
